# Early clinical exposure in undergraduate dental education: a systematic review of effectiveness, experiences, and outcomes

**DOI:** 10.1186/s12909-026-08692-z

**Published:** 2026-01-28

**Authors:** Amol R. Gadbail, Shailesh M. Gondivkar, Monal Yuwanati, Archana Sonone, Mithilesh Dhamande, Aarti Panchbhai, Sachin C. Sarode

**Affiliations:** 1 Department of Dentistry, Government Medical College and Hospital, Nagpur, Maharashtra 440003 India; 2 Department of Oral Medicine & Radiology, Government Dental College & Hospital, Nagpur, Maharashtra India; 3 Department of Oral Pathology and Microbiology, Saveetha Institute of Medical and Technical Sciences, Saveetha Dental College and Hospital, Saveetha University, Chennai, Tamil Nadu India; 4 Department of Oral Pathology and Microbiology, Datta Meghe Institute of Higher Education & Research (DMIHER), Sharad Pawar Dental College and Hospital, Sawangi (M), Wardha, Maharashtra India; 5 Department of Prosthodontics, Crown and Bridge, Datta Meghe Institute of Higher Education & Research (DMIHER), Sharad Pawar Dental College and Hospital, Sawangi (M), Wardha, Maharashtra India; 6 Department of Oral Medicine and Radiology, Datta Meghe Institute of Higher Education & Research (DMIHER), Sharad Pawar Dental College and Hospital, Sawangi (M), Wardha, Maharashtra India; 7 Department of Oral Pathology and Microbiology, Dr. D.Y. Patil Vidyapeeth, Dr. D.Y. Patil Dental College and Hospital, Pune, Maharashtra India

**Keywords:** Clinical exposure, Dental education, Undergraduate, Effectiveness, Professional identity, Communication

## Abstract

**Purpose:**

The objective of this systematic review was to synthesize evidence on the early clinical exposure (ECE) and its effectiveness, student experiences, and outcomes in undergraduate dental education.

**Methods:**

In August 2025, the systematic search was carried out in PubMed, Scopus, and Web of Science. The quantitative, qualitative, and/or mixed-methods peer-reviewed studies evaluating ECE to undergraduate dental students were included. Summary synthesis of included studies was carried out. The risk of bias and quality assessment was assessed with Cochrane Risk of Bias 2, ROBINS-I tool, Joanna Briggs Institute, and Critical Appraisal Skills programme.

**Results:**

Nine studies (2007- August 2025) from a range of countries were included. ECE interventions were chairside demonstration, peer-assisted shadowing, simulation, clinical/community placements, and hybrid models. There is evidence of effectiveness of ECE in knowledge, psychomotor skills, and affective domains. In addition, student experiences were reported as positive and significant increases in satisfaction, confidence, motivation, clinical reasoning and other learning gains such as professional identity, empathy, and communication. Six out of nine included studies were non-randomized studies. All the non-randomized studies were reported methodological biases, including small single-center samples, self-report measures, short follow-up periods, and rated as some concern and high risk of bias.

**Conclusions:**

ECE in undergraduate dental education promising contribution to the cognitive, psychomotor, and affective domains and reduces transition stress to clinical training. However, presence of methodological bias in included studies undermines the evidence. Future studies with robust objective performance measures, multicentre designs, and longitudinal follow-up should be carried out to better establish the educational and clinical impact of ECE in dental education.

## Introduction

The transition from preclinical education to clinical training is a critical and sometimes troublesome time in health sciences education. Across all disciplines, students typically find it difficult to connect theoretical concepts to actual patients. Students experience increased anxiety and identify themselves as not being prepared for their clinic responsibilities. Early clinical exposure (ECE) is one curricular approach that bridges this gap by providing real clinical experience through simulation, shadowing, community placements, or supervised patient interactions early in preclinical training [1, 2].

ECE is theoretically based on Kolb’s experiential learning theory, focusing on learning through experience, reflection, and application in a clinically authentic manner. Situated learning and professional identity formation view ECE as opportunities to enter early into clinical communities of practice, thus developing legitimate peripheral participation and gradual development of professional roles and values [3].

ECE is widely established in the medical education across the globe [1]. Various studies on ECE in medical education emphasizes better retention of knowledge, communication skills, empathy, professional identity, and fewer signs of transition stress and greater motivation [1, 2, 4]. High-fidelity simulation-based ECE in nursing builds student confidence. It promotes collaborative inter-professional working relationships. It also enhances psychomotor competence and improves integration of theoretical knowledge with clinical practice [5]. The same advantages are also seen in the allied health profession of physiotherapy as early patient involvement enables the development of skills and invites patient-centered values [6].

In contrast, dental education has been traditionally dependent on extended preclinical laboratory training prior to student exposure to the clinic [7]. Although, dental curricula have offers training and technical skill instruction on mannequins or typodonts, it is not unusual to forego patient contact until the 3rd or 4th year of education. Under this model, preclinical instructions are disconnected from actual application, amplifying anxious feelings while transitioning to clinic and various dental operating procedures on actual patients. Few dental schools currently incorporate ECE activities. These may include chairside patient demonstrations, peer-assisted shadowing, and community outreach projects. Some programs also use hybrid models that combine simulation experiences with supervised care of real patients [8].

The latest evidence indicates that ECE in dentistry generates analogous results to medical and other allied health sciences education [8]. However, most of the available evidence remains single-center in nature, heterogeneous in the design of the intervention, often descriptive, and studies comparing ECE approaches or their effect on measurable educational outcomes are limited. In contrast to medical and nursing, which have strongly defined ECE on a substantial body of evidence due to multi-centre studies with large cohorts, and longitudinal evaluations, dental education has no such robust evidence base particularly long-term retention of skills and patient-centered outcomes.

Even with the increasing curricular reforms focused on competency-based education, earlier patient exposure and simulation technologies enacted into curricula, no recent systematic review synthesizes the effectiveness and educational impact of ECE specifically for undergraduate dental students. This is an important knowledge gap given that dental educators might have little synthesized evidence to guide the design, implementation, and evaluation of ECE within dental curricula.

To address this gap, the present systematic review aims to summarize published evidence on ECE in dental undergraduate education for three purposes: (i) to evaluate its effectiveness in improving knowledge, skills, clinical reasoning, and performance, (ii) to evaluate students’ and teachers’ experience, satisfaction, confidence, and perceived barriers, and (iii) to evaluate broader educational outcomes related to professional identity, communication, empathy, and teamwork.

## Materials and methods

### Protocol and reporting standards

The above systematic review is reported according to the Preferred Reporting Items for Systematic Reviews and Meta-Analyses (PRISMA) (2020) statement [9]. The protocol for the review was prospectively registered with the International Prospective Register of Systematic Reviews (PROSPERO) with registration number CRD420251142350.

### Eligibility criteria

The studies were considered eligible if

i) Studies conducted on undergraduate dental students;
ii) Studies evaluating ECE such as classroom-based exposure, hospital/clinical-based exposure and community-based exposure;
iii) Studies assessed outcomes including knowledge, psychomotor skills, clinical reasoning, objective assessments performance, confidence, professional identity, communication, empathy, and learner and faculty experiences/feedback;
iv) Quantitative (randomized controlled trials, quasi-experimental, cohort design, or cross-sectional survey), and qualitative or mixed-methods studies;
v) Only peer-reviewed full-text papers published in English were included in the review, with no restrictions on the year of publication and location.

Studies that included postgraduate or continuing dental education, editorials, commentaries, consensus documents, conference abstracts were excluded. In addition, we excluded short reports, opinion papers, commentary pieces, surveys, and narrative review.

### Information sources and search strategy

A systematic literature search was carried out in the PubMed/MEDLINE, Scopus, and Web of Science databases for articles published up to August 2025 from their inception. In addition, cross reference of selected articles was screened to identify additional articles. The detailed search strategy was mentioned in Table 1.

**Table 1:** Search string results from major databases

Database	Keyword string	Total hits
Pubmed	(“early clinical exposure“[tiab] OR “early patient contact“[tiab] OR “preclinical exposure“[tiab] OR “early clinical experience“[tiab] OR “early clinical placement“[tiab]) AND (“dental students“[tiab] OR “undergraduate dental“[tiab] OR “dental curriculum“[tiab] OR “dental training“[tiab] OR dental[tiab])	16
Scopus	TITLE-ABS-KEY(“early clinical exposure” OR “early patient contact” OR “preclinical exposure” OR “early clinical experience”) AND TITLE-ABS-KEY(“dental students” OR “undergraduate dental” OR “dental curriculum” OR “dental training” OR dental)	24
Web of science	TS=(“early clinical exposure” OR “early patient contact” OR “preclinical exposure” OR “early clinical experience”) AND TS=(“dental students” OR “undergraduate dental” OR “dental curriculum” OR “dental training” OR dental)	10

### Study selection

**Fig. 1:**
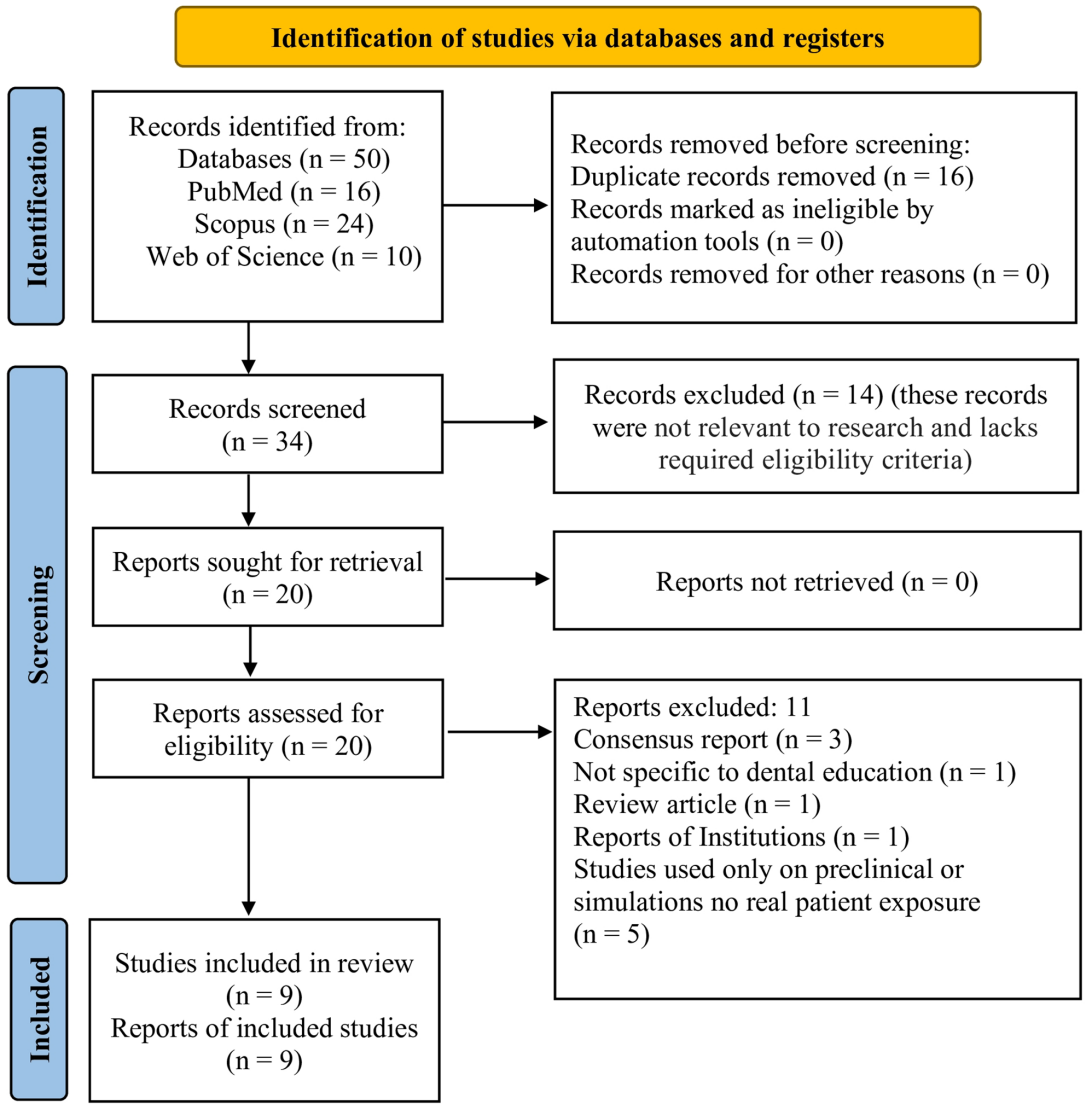
Screening and selection flow chart according to PRISMA guidelines

Upon removal of duplicates, the titles and abstracts of all the records retrieved were independently screened by AG & SG against the pre-defined eligibility criteria. Full text articles identified as potentially relevant were retrieved and assessed for inclusion. Any disagreements between the assessors were resolved by discussion with senior assessor (SS). The reasons for exclusion and details of the selection process at the full-text level were clearly documented and presentedin a PRISMA 2020 flow diagram, to enhance the transparency and reproducibility of the study (Fig. 1).

### Data extraction

Data from the included studies were extracted independently by two reviewers (AG and SG) using a predefined data extraction sheet and any disagreements were resolved by consensus. The variables extracted from studies included: bibliographic details (author, year of publication, country), study design, methodological characteristics, sample size, academic level of student, information on the ECE intervention (type, timing, duration, and delivery mode), comparator (if appropriate), and the outcomes measured, including knowledge, psychomotor skills, clinical reasoning, objective assessments performance, confidence, professional identity, communication, empathy, and learner and faculty experiences/feedback. Additionally, we recorded data on the assessment tools used and the methods of validation for each study.

### Risk of bias assessment

The risk of bias and/or methodological quality of each included study’s data was assessed by two independent reviewers (AG & MY) using validated instruments based on study design. Randomized controlled trials were evaluated with the Cochrane Risk of Bias 2 (RoB 2) tool [10], whereas non-randomized studies were assessed using the Risk of Bias in Non-randomized Studies of Interventions (ROBINS-I) tool [11]. Cross-sectional studies were evaluated using the Joanna Briggs Institute (JBI) critical appraisal checklist [12] and qualitative studies were assessed using the Critical Appraisal Skills Programme (CASP) qualitative checklist [13]. Risk of bias for each study was rated as the criteria of the specific tool. Disagreements between reviewers were resolved through discussion with a third reviewer (SS).

### Summary synthesis

In light of expectation for heterogeneity in the study designs, interventions, and outcome measures, meta-analysis was unfeasible and a summary synthesis without meta-analysis was planned. Quantitative data including knowledge test scores, OSCE/OSPE performance, and self-reported level of confidence were described and compared when relevant. Qualitative data about student and faculty experiences/feedback were then synthesized in a thematic way using an inductive coding strategy. Quantitative and qualitative results were then synthesized together to create a holistic sense of the effectiveness, experiences, and outcomes of ECE in dental education for undergraduates.

## Results

### Study selection

The electronic search of PubMed, Scopus, and Web of Science yielded 50 records. Following the removal of duplicates, 34 titles and abstracts of records were screened. Out of which 14 were excluded as these records were not relevant to research and lacks required eligibility criteria. The remaining 20 full texts records were assessed for eligibility. Finally, nine studies fulfilled the inclusion criteria and were considered in the final synthesis. The process of study selection is illustrated in the PRISMA 2020 flow diagram (Fig. 1).

### Characteristics of included studies

#### Study settings and populations

Nine studies between the years 2007 and August 2025 were included with a great variety of institutions and geographic locations. One third of studies (*n* = 3) were from India [7, 8, 14]. The remaining studies comprised one each from United States of America (*n* = 1) [15], Germany [16], Denmark [17], Pakistan [18], Chile [19], and Trinidad and Tobago [20]. Sample sizes varied from small qualitative groups (*n* = 12) [17] to large group (*n* = 163) [15]. The participants were preclinical undergraduate students (1st and 2nd year), while some were clinical (3rd year) and final year participants [17, 19]. The participation of faculty was used in studies, with universities employing the participation of faculty as supervisors or facilitators [7, 16], though some studies used peer-assisted learning models, where senior students mentored junior students [8] (Table 2).

**Table 2:** Characteristics of included studies

Sr No.	Author-Year	Country/Setting	Study Design	Sample Size	Student Level	Type of ECE	Timing	Duration/Frequency	Mode of Delivery
1	Ali et al., 2025 [18]	Pakistan; HBS Dental College, Islamabad	non-randomized Comparative quasi-experimental cross-sectional survey (two groups).	150 students (75 Group A EPCE, 75 Group B late exposure).	2nd-year BDS students.	Early preclinical exposure with structured patient interaction, OSCE stations, and questionnaire-based evaluation.	2nd year, during preclinical phase before transition to clinical postings.	One semester; multiple OSCE stations and structured placements.	clinical/hospital-based on Real and standardized patients;
2	Shettigar et al., 2025 [14]	India; Manipal College of Dental Sciences, Mangalore (Manipal Academy of Higher Education)	Randomized controlled educational study.	65 students (Control: 29; ECE: 36).	Second-year undergraduate dental students (preclinical prosthodontic).	Hybrid, didactic + preclinical phantom head training + clinical video demonstration of primary impression procedure.	Second year (preclinical prosthodontics).	One structured ECE module integrated into prosthodontics practical course.	Simulation-based Video demonstration on real patient + phantom head practice with alginate impressions.
2	Maheshwari et al., 2024 [7]	India; ESIC Dental College and Hospital, New Delhi (single-center)	Non-randomized prospective educational interventional study (pre-post single group with mixed-methods).	Students *n* = 50 (complete enumeration of eligible 2nd-year students); Patients *n* = 5 (Class I edentulous patients).	BDS 2nd year (pre-clinical prosthodontic).	Chairside ECE: clinical demonstration on real patients (complete denture border molding and jaw relation). plus prior didactic and lab sessions; MCQ tests used for assessment.	Second year (pre-clinical), after completion of didactic and lab sessions.	Conducted over 4 months (July to Oct 2022); students divided into 5 groups of 10 for demonstrations; post-test 1 week after ECE sessions.	clinical/hospital-based on Real patients
4	Tricio et al., 2024 [19]	Chile; Universidad de los Andes, Santiago	Randomized controlled educational trial with digital grading.	68 (34 intervention, 34 control).	Fourth-year undergraduate dental students (starting clinical practice).	Simulation with patient-specific 3D-printed teeth, followed by patient treatment.	Fourth year, at transition from preclinical to clinical.	One preparatory session before clinical treatment.	Simulation-based followed by real patient exposure
5	Engel et al., 2024 [15]	USA; Harvard Medical School, Boston, MA	non-randomized Pilot mixed-methods program evaluation (convergent mixed-methods)	163 first-year medical and dental students participated; 127 surveys returned (78%); 124 analyzed; 109 completed both survey parts.	First-year medical and dental students (pre-clinical).	Clinical shadowing (mentored early clinical exposure) across inpatient, outpatient, and home visit hospice/palliative care settings.	First year (spring 2022), during preclinical clinical skills course.	Single half-day clinical immersion session per student (part of a 10-week clinical immersion series).	clinical/hospital-based Real patient encounters
6	Hande et al., 2023 [8]	India; Sharad Pawar Dental College and Hospital, Wardha, Maharashtra	Randomized controlled educational intervention (experimental vs. control with peer-assisted vs. faculty-assisted ECE).	125 students total: 100 1st BDS (50 Group A experimental, 50 Group B control), 25 2nd BDS high-achievers as peer instructors.	1st-year BDS students (pre-clinical Oral Histology); 2nd-year BDS acted as peer instructors.	Peer-assisted clinical demonstrations on patients integrated with histology concepts.	1st-year BDS, after didactic lectures and practicals in Dental Histology.	6-month intervention; ratio peer: student 1:2; both groups exposed to OMM ECE module.	Peer shadowing Real patients (clinical demonstrations), peer explanations, small group teaching.
7	Ramlogan and Raman, 2022 [20]	Trinidad and Tobago; School of Dentistry, University of the West Indies	non-randomized Educational intervention study with intra-individual analysis (formative assessment study).	55 third-year dental students (2016 *n* = 27, 2017 *n* = 28).	Third-year DDS students at start of clinical training (introductory clinical periodontology).	Chairside clinical tasks with self- and staff-assessment, reflections.	Third year (first clinical semester).	Three educational sessions within one month.	clinical/hospital-based on Real patients (peer-as-patient), phantom-head demos, clinical checklists, videos, lectures.
8	Moore et al., 2021 [17]	Denmark; Aarhus University Dental School	non-randomized Qualitative descriptive study with semi-structured interviews (*n* = 12) and a validation true/false questionnaire (*n* = 12); mixed-methods validation.	12 final-year female dental students (purposive sample); validation questionnaire completed by same 12.	Final year students (9th − 10th semester); had experience as mentees (1st − 4th semesters) and mentors (7th − 10th ).	Mentee/mentor early clinical experience (assistantship/shadowing/assisting with real patients in teaching clinic). (suction assistance, data entry, basic tasks; occasional simple procedures).	Mentee activities during 1stâ€“4th semesters; mentorship roles in later semesters (5th − 10th as applicable).	FOAL program requires 12 h per semester during 1st − 4th semesters; program ongoing since 2012 in current form.	Peer shadowing on Real patient exposure in teaching clinic
9	Ratzmann et al., 2007 [16]	Germany; University of Greifswald	non-randomized quantitative, pre–post, questionnaire-based evaluation Program description with summative student evaluation	*n* = 108 participants (evaluations across first four academic years)	Pre-clinical students (first 2 years; first four semesters)	Community-oriented early patient contact (home visits, patient consultations, seminars, POL tutorials)	Implemented during first four semesters (pre-clinical phase)	Visits once a month over 1 year per student; course spans four semesters (two years)	Community placement Real patients

*OSCE *Objective structured clinical examination, *EPCE *early preclinical exposure, *BDS *bachelor of dental surgery, *ECE *early clinical exposure, *OMM *oral mucous membrane, *DDS *Doctor of Dental Surgery, *POL *problem-oriented learning

Across all nine studies, multiple approaches were employed to design methods to examine ECE. Three studies conducted randomized controlled trials to compare ECE interventions versus traditional instruction [8, 14, 19]. Various other studies used quasi-experimental or pre-post intervention designs that included multiple-choice questions (MCQ), objective structured clinical exams (OSCEs), and clinical performance checklists to assess learning outcomes [7, 18, 20]. One study used cross-sectional survey designs to explore student perceptions of ECE [16], and two studies used a mixed-methods design with interviews, reflections, and questionnaires to study the experiences of learners in a shadowing-based ECE model [15, 17]. As a group, these nine studies demonstrate a reasonable mix of experimental, quasi-experimental, survey based, and mixed methods designs in undertaking investigations related to ECE in undergraduate Dental Education.

### Types of ECE interventions and mode of delivery

Across the studies, we noted that the ECE was implemented using three types of delivery such as clinical/hospital-based, simulation-based, community-based and peer/professional shadowing. Clinical ECE primarily consisted of chairside demonstrations and supervised contact with ‘real live patients’ in prosthodontics, oral medicine and periodontology [7, 8, 20]. Several of the studies incorporated simulation-based delivery, standardized patients, and video of clinical procedures before working with real patients [14, 19]. Shadowing and community-based delivery enabled junior students to observe their senior peers or department-based supervised clinical immersion [15, 17]. A number of programs also provided preclinical to clinical bridging modules that connected early patient exposure to more formal assessment such as OSCE [18]. In conclusion, collectively these types of different delivery modes reveal that there are structured and varied approaches to exposing students to real patient care during their dental education (Table 2).

### Duration and timing

Duration and frequency of the interventions were quite diverse. Some were short-intervention that included a brief intervention of several hours in the style of half-day shadowing or skill-building session [20] and other lasted a semester or several years [16]. Timing of program initiation differed. Some programs commenced in the 1st year, as a way of integrating basic sciences and clinical learning [18], while others started their formal program in the 2nd or 3rd year as lead into clinical training [7, 8] (Table 2).

### Comparators and controls

Most employed pre and post evaluation designs with no controls [7, 16], thus excluding the possibility of results being caused by ECE only. Hande et al. [8] compared peer-assisted and faculty-assisted ECE and had higher post-test knowledge and OSPE scores in the peer-assisted group. Tricio et al. [19] compared traditional patient care with simulation-based interventions and also recorded an increased competence among students when employing patient-specific 3D-printed models. These studies were still limited by their single-center setting and short-term follow-up, though these designs were of higher quality.

### Effectiveness outcomes

The nine studies of ECE clearly showed positive impact in effectiveness measures, across changes in knowledge, clinical skills, and performance. Several of studies assessed significant improvements in MCQ and written tests scores [7, 14], and better performance on the OSPE or OSCE [8, 18], and improved accuracy on clinical tasks such as crown preparation and impression making [19]. Other studies measured improvement in professionalism, evaluative judgement, and use of theoretical knowledge in practice [16, 20]. Evidence from the shadowing and immersion experiences extended on students understanding of the clinical workflow of real-life practitioners and provided opportunities to prepare for patient interaction [15, 17]. Overall, these findings reflect ECE is effective for enhancing core cognitive, psychomotor, and professional attributes in undergraduate dental students (Table 3).

**Table 3:** Effectiveness, experiences, and educational outcomes

Sr. No.	Author-Year	Effectiveness Outcomes (Knowledge/Skills/Performance)	Experiences (Satisfaction/Confidence/Challenges)	Educational Outcomes (Identity/Empathy/Teamwork)
1	Ali et al., 2025 [18]	Group A showed significantly higher academic performance (theory mean 82% vs. 76%, *p* = 0.002, d = 1.55), OSCE communication (85% vs. 78%, *p* = 0.004, d = 2.15), empathy (83% vs. 75%, *p* = 0.005, d = 2.28).	Self-reported confidence higher in EPCE group (84% vs. 77%, *p* < 0.005). 90% of Group A reported feeling very confident vs. 70% in Group B.	EPCE improved communication, empathy, teamwork, and readiness for clinical practice.
2	Shettigar et al., 2025 [14]	ECE group showed significantly higher post-test MCQ scores (8.64Â±1.42 vs. 6.69Â±0.85, *p* < 0.001) and improved OSPE scores in tray selection, mixing, impression quality, loading/orientation, and recording of landmarks (all *p* < 0.001).	Student feedback strongly positive 85.7% strongly agreed and 14.3% agreed that ECE improved understanding; students felt more motivated and recommended expanding ECE modules.	Improved visualization and comprehension of impression procedures; enhanced psychomotor skills and motivation for learning.
3	Maheshwari et al., 2024 [7]	Significant improvement in MCQ mean scores: Overall pre 42.96Â±11.00 vs. post 54.08Â±10.09 (*p* = 0.001); FI pre 22.88–28.52 (*p* = 0.001); JR pre 20.08–25.56 (*p* = 0.001).	High student satisfaction; satisfaction indices > 90 for most items; students reported increased interest, understanding, retention, confidence; 100% preferred inclusion of ECE in PCP curriculum.	Students reported improved confidence and anticipated better communication with patients; faculty noted increased faculty-student interaction and professional development.
4	Tricio et al., 2024 [19]	Adequate taper: 43.5% vs. 25.7% (*p* < 0.001); Less excess reduction: 33.6% vs. 49.3% (*p* = 0.011); Adequate occlusal clearance: 51.9% vs. 43.0% (*p* = 0.044).	Higher self-confidence in intervention group (*p* < 0.001); 70.6% strongly agreed practice improved preparedness; 76.5% disagreed it was not worth time.	Better preparedness, reduced anxiety, improved autonomy.
5	Engel et al., 2024 [15]	Post-experience Likert results: 98% reported increased understanding of how palliative care enhances patient care; 83% reported interacting with interdisciplinary teams; 77% strongly agreed they could envision how palliative care complements care; increased confidence reported (39% strongly agree, 40% agree).	High positive reactions: adjectives skewed positive (hopeful, inspiring, eye opening); many reported emotional impact but felt supported; 67% indicated increased interest in palliative care; students reported increased confidence and reflection.	Reported gains in understanding interdisciplinary teamwork, patient-centered communication, attention to psychosocial/spiritual needs, empathy development, and professional identity formation.
6	Hande et al., 2023 [8]	Group A (peer-assisted) showed significantly higher post-test (7.46 Â±1.32) vs. Group B (3.98 Â±1.59, *p* < 0.001). OSPE scores also higher in Group A (4.12 Â±1.00) vs. Group B (2.94 Â±1.23, *p* < 0.001).	90% strongly agreed peer-assisted ECE was valuable; 86% strongly agreed it should be integrated into curriculum; feedback highlighted better retention, integration, and peer support.	Improved peer bonding, confidence, communication, teamwork, and understanding of clinical application of preclinical knowledge.
7	Ramlogan and Raman, 2022 [20]	Medium correlations (*r* = 0.32 cognitive; *r* = 0.44 non-cognitive) between student and staff; small correlations with written exam (*r* = 0.22–0.29 students, *r* = 0.31–0.34 staff). Students mean scores improved over sessions; professionalism significantly improved (*p* = 0.000).	Students accepted self-assessment, valued feedback and reflection; recognized need for clear guidelines and practice to improve accuracy.	Improved evaluative judgement, communication, teamwork, professionalism, reflective practice, and readiness for independent practice.
8	Moore et al., 2021 [17]	Perceived partial reduction in preclinicalâ€“clinical transition stress, improved communication skills, increased motivation, observational learning aiding competence development.	Overall positive perception; reported benefits (motivation, role models, social integration); challenges included lack of organization, insufficient clinical hours, anxiety and lack of basic clinical knowledge.	Enhanced professional identity, communication skills, self-efficacy, collaborative skills and mentor learning-by-teaching benefits.
9	Ratzmann et al., 2007 [16]	Students reported improved understanding of community dentistry, relation between general and dental diseases, and ability to apply theoretical knowledge in practice; POL usefulness rated positively	Majority supported EPC at curriculum start (78.7%); satisfaction with consultations (65.8%); mixed responses on psychological skills and self-assessment	Enhanced communication skills, patient-centered perspective, motivation, professional identity development

*OSCE *Objective structured clinical examination, *EPCE *early preclinical exposure, *ECE *early clinical exposure, *OSPE *Objective structured practical examination, *FI* final impression, *JR *jaw relation, *PCP *pre-clinical prosthodontics, *EPC *Early Patient Contact

### Student/learners experiences and educational outcome

The nine studies indicated that learners overwhelmingly reported positive experiences with ECE, describing increases in confidence, motivation and comfort while in patient contact. ECE contributes to meaningful improvements in professional identity, communication, empathy, and clinical preparedness for clinical practice. Students expressed benefits to having early exposure to real clinical environments, greater ease in transitioning to clinical training and greater relevance of their course work [7, 16, 17]. Learners expressed satisfaction in ECE experiences that supported simulation, peer-assisted, and early preclinical exposure [8, 14, 18], while benefiting from shadowing and interdisciplinarity, both of which enhanced reflective learning and emotional preparation for patient care [15, 19, 20]. Overall, the student experience strongly endorsed ECE as a formative approach to early learning. In all, ECE had a number of consistently positive effects as education outcomes on early professional development (Table 3).

### Faculty feedback

Faculty opinions, although limited in number were positive overall. Maheshwari et al., (2024) [7] noted high levels of satisfaction for prosthodontic faculties who appreciated increased student readiness but felt time pressures. Faculty provided community-based visits in another study and highlighted the need for formal supervision [16] (Table 4).

**Table 4:** Quantitative, qualitative results and faculty/patient feedback

Sr. No.	Author-Year	Key Quantitative Results	Key Qualitative Themes	Faculty Feedback
1	Ali et al., 2025 [18]	Theory exam: 82% vs. 76% (*p* = 0.002, d = 1.55); Communication: 85% vs. 78% (*p* = 0.004, d = 2.15); Empathy: 83% vs. 75% (*p* = 0.005, d = 2.28); Confidence: 84% vs. 77% (*p* < 0.005, d = 2.00).	EPCE enhances knowledge acquisition, empathy, confidence; reduces anxiety; bridges gap between theory and practice.	No formal patient feedback; faculty trained and standardized OSCE assessments.
2	Shettigar et al., 2025 [14]	MCQ pre/post: Control 5.41 (± 6.69), ECE 7.31 (± 8.64) (*p* < 0.001). OSPE: tray selection 3.83 vs. 2.71; mixing 2.82 vs. 1.91; impression quality 2.43 vs. 1.50; loading/orientation 2.5 vs. 1.47; landmarks 3.15 vs. 2.16 (all *p* < 0.001).	ECE bridges gap between theory and practice, increases motivation, and improves correlation of theoretical and practical skills.	Faculty prepared and validated teaching video; patient consent obtained for recording; no direct patient feedback collected.
3	Maheshwari et al., 2024 [7]	All 50 students completed study; mean age 21.38 (± 0.90); female 66%; overall mean score increase from 42.96 to 54.08 (*p* = 0.001); satisfaction indices range 87.8–97.6; paired t-test used.	Faculty satisfaction; beneficial for students & patients; faculty shortage; time-consuming; difficult timetable integration; increased interaction; suggestion of video-assisted teaching.	Faculty interviews (*n* = 2) showed satisfaction but raised concerns: faculty shortage, time-consuming, integration into timetable; patients received CDs at study end.
4	Tricio et al., 2024 [19]	Adequate taper ↑, excess reduction ↓, clearance ↑; confidence ↑ significantly (*p* < 0.001).	3D models realistic, increased confidence, reduced anxiety; softer than enamel but useful bridge.	Faculty validated digital assessments; no patient feedback collected.
5	Engel et al., 2024 [15]	127 surveys returned (78%); Part B completed by 109 (67%). Example items: ‘Understand how palliative care could complement care 77% strongly agree; ‘Articulate differences between palliative and hospice care 50% strongly agree; 98% reported increased understanding of role of palliative care; 67% reported increased interest in HPM.	Three overarching themes: operational dimensions (care spectrum, teams, symptom management), psychosocial dimensions (patient-centered communication, non-medical care), and personal impact/self-reflection (emotional impact, discordance of expectations, inspiration to learn).	Faculty provided feedback for program evaluation (not part of study analysis); no formal patient feedback collected.
6	Hande et al., 2023 [8]	Pre-test scores: 3.26 vs. 3.34; Post-test: 7.46 vs. 3.98 (*p* < 0.001); OSPE: 4.12 vs. 2.94 (*p* < 0.001); 90% valued PAL-ECE; 86% supported integration into curriculum; 80% rated outstanding tool.	Peer-assisted ECE enhances motivation, learning, integration, psychological support; students suggested broader application to other topics.	Faculty supervised but patient feedback not reported; peers noted enhanced teaching skills.
7	Ramlogan and Raman, 2022 [20]	Cognitive mean *r* = 0.27 raw (0.32 corrected), non-cognitive mean *r* = 0.37 raw (0.44 corrected); significant gains in professionalism (*p* = 0.000); no significant differences between student and staff mean scores ( *p* > 0.05).	Self-assessment feasible in early clinical training; improved over time; students tended to emphasize non-cognitive items; guidance and feedback critical for accuracy.	Faculty validated assessments and provided feedback; no direct patient feedback reported.
8	Moore et al., 2021 [17]	Validation questionnaire results (Table 3): e.g., item 11 (FOAL helps transition) 75% true; consensus = 0.94; mean competence 0.74; sample adequacy analyses in Table 4.	Overall positive but needs improvement; advantages as mentee and mentor; challenges (lack of introduction, training, organization); inspiration/reaffirmation; suggestions for better scheduling and increased hours.	Not empirically collected; program includes mentor/teacher involvement but no formal faculty/patient feedback reported.
9	Ratzmann et al., 2007 [16]	*n* = 108; 78.7% supported EPC at start; 76.9% disagreed EPC should be after pre-clinical exams; various tabled item-level frequencies (Tables 1â€“4)	Positive motivation, improved patient understanding, success of POL seminars albeit mixed on future application; organizational/time/resource challenges	Faculty supervised visits; patient selection aided by staff; no formal patient feedback reported

*OSCE *Objective structured clinical examination, *EPCE *early preclinical exposure, *ECE *early clinical exposure, *OSPE *Objective structured practical examination, *CD* Complete Denture, *HPM *hospice and palliative medicine, *PAL *peer-assisted learning, *EPC *Early Patient Contact, *POL *problem-oriented learning

### Assessment techniques

The evaluation of ECE outcomes in the studies differed substantially. The quantitative measures employed validated MCQ tests [7] and OSPE/OSCE examinations [8, 18]. The qualitative studies employed semi-structured interviews [17]. The community-based interventions evaluated outcomes via summative questionnaires [16] and the simulation-based intervention evaluated using structured rubrics [19].

### Reliability and validity

The studies reported on reliability and validity in various ways. Although about half of the studies gave clear evidence of validation of their tools [7, 8, 17, 20], other studies gave little or no evidence of validation of the instrument. Ratzmann et al., (2007) [16] employed routine evaluation tools but indicated no application of any psychometric validation. This variety of methodology and degree of rigor shows the necessity of employing standardized and validated outcome measures in ECE in dental education research for the sake of study comparability and enhancing confidence in results.

### Risk of bias

Out of nine, three studies were rated as high risk of bias, where as six studies were rated as some concern on account of key methodology factors, including small sample sizes, single-center studies, and limited control for confounders. Three randomized control trials were rated as some concern [8, 14, 19] by using RoB 2.0. The quasi-experimental and non-randomized studies were rated as some concern to high risk, primarily for lack of randomization, lack of controls, and because they relied on self-reported outcomes [7, 18, 20] by using ROBINS-I and AXIS tool. Qualitative and mixed-method studies were rated as some concern risk primarily because of small purposive samples, which limited generalizability [15, 17] by using CASP and JBI Critical Appraisal tools. The cross-sectional program evaluation also indicated high risk primarily because it relied on self-report measures and no comparators were utilized [16] by using the ROBINS-I tool. Overall, the evidence on the effectiveness of ECE in dental education is limited due to variations in methodological rigor and the potential bias (Table 5).

**Table 5:** Risk of bias assessment, strength reported and limitations reported

Sr No.	Author-Year	Risk of Bias Tool	Risk Judgment	Strengths Reported	Limitations Reported
1	Ali et al., 2025 [18]	AXIS (program evaluation) (non-randomized studies of interventions).	Some concern (non-randomized, potential confounders, self-reported measures).	Large sample (*n* = 150), validated tools, OSCE structured assessments, strong statistical significance with effect sizes.	Single-institution, non-randomized, potential self-selection bias, self-reported outcomes, short-term follow-up.
2	Shettigar et al., 2025 [14]	RoB 2.0 (Cochrane Risk of Bias for RCTs).	Some concern (randomized, validated tools, but single-center, short-term).	RCT design, validated tools, significant improvement in cognitive and psychomotor skills, positive student perceptions.	Single institution, small sample, one exercise only, limited generalizability.
3	Maheshwari et al., 2024 [7]	ROBINS-I (for non-randomized interventions)	High risk of bias (non-randomized, no control, potential selection bias with purposive sampling, single-center, small sample).	Prospective interventional design, mixed-methods (quant + qual), validated assessment tools, full participation, practical patient-based demonstrations.	Single-center, single cohort, no control group, short-term outcomes only, small patient number, generalizability limited; long-term skill retention not assessed.
4	Tricio et al., 2024 [19]	RoB 2.0 (Cochrane RCT). randomized control trial	Some concern (sequence generation and allocation concealment not described, Perception questionnaires are subjective and not blinded.)	RCT, objective digital grading, validated questionnaires.	Single site, small sample, limited generalizability, models not identical to enamel.
5	Engel et al., 2024 [15]	JBI Critical Appraisal Checklist for Quasi-Experimental / Pre-post studies	Some concern (single-center program evaluation, no control, self-reported outcomes, decent response rate and qualitative rigor).	Large cohort for pilot (*n* = 163), mixed-methods convergent analysis, high response rate (78%), rigorous qualitative coding with codebook and consensus, diverse clinical settings.	Single institution, single short exposure (half-day), variable site experiences, no control/comparator, limited longitudinal follow-up, self-report survey, possible response bias.
6	Hande et al., 2023 [8]	RoB 2 (randomized trials)	Some concern (randomized but small single-center study; potential performance bias with peer selection).	Randomized design, control group, peer role models, multiple assessment methods.	Single-center, small sample size, short duration, limited generalizability; patient feedback absent.
7	Ramlogan and Raman, 2022 [20]	ROBINS-I	High risk of bias (educational intervention, no randomization, small sample).	Innovative intra-individual approach; separation of cognitive/non-cognitive items; validated instruments; correction for attenuation; formative methodology.	Single-institution, small sample size, no sampling strategy, short study duration, did not examine gender/culture effects.
8	Moore et al., 2021 [17]	CASP Qualitative Checklist	Some concern (appropriate design but small purposive sample and limited transferability).	Rigorous qualitative methods, purposive sampling for perspectives, use of NVivo, validation questionnaire with consensus analysis, ethical approval.	Small purposive sample (*n* = 12), all female, single-center, findings may not generalize; no objective stress measures; potential insider bias.
9	Ratzmann et al., 2007 [16]	ROBINS-I	High risk of bias (program evaluation with self-reported student questionnaires, no control/comparator)	Longitudinal program over 4 years, systematic student evaluation, integration with community medicine and POL	Resource/time intensive; uncertain long-term impact on clinical preparedness; evaluations based on self-report; no control group

*OSCE *Objective structured clinical examination, *RCT *randomized control trial

### Quality assurance

During screening and data extraction, inter-rater reliability was assessed. At the pilot phase, agreement was high with a kappa at title/abstract screening of κ = 0.82 and a kappa at full-text screening of κ = 0.79, effectively providing a substantial level of reliability. For data extraction agreement remained robust (κ = 0.81) and any disagreement was clarified through consensus meetings.

## Discussion

In spite of the diversity of study designs and settings, the overarching similarity among the studies was that ECE positively changed students’ knowledge, clinical competence, confidence, professional identity, and overall readiness to deliver patient care. These results should be interpreted cautiously given the heterogeneity of study designs, outcome measures, and the predominance of single centre studies with short follow-up periods. Overall, the studies yield evidence that ECE enhances Bloom’s taxonomy on the cognitive, psychomotor and affective domain, contributing to the multi-dimensional learning outcomes, which are the cornerstone of competency-based dental education.

In the studies included, ECE was provided through four broad categories or modalities: clinical/hospital-based [7, 15, 18, 20], simulation-based [14, 19], community-based [16] and peer/professional shadowing [8, 17]. Clinical ECE often involved chairside observation and supervised facing patient contact, linking theoretical learning experiences with real procedures early in training [7, 8, 20]. Simulation-supported activities provided opportunities to develop initial procedural and communication skills in a controlled environment before engaging actual patients [14, 19]. Similarly, these mirroring approaches widely adopted in medical and nursing education to support early skills acquisition and theory to practice integration [21, 22]. Shadowing and community-based placements extended exposure further by facilitating students to observe senior peers, to participate in supervised immersion, and to experience various patient settings [15–18]. These findings were consistent with evidence from medicine and nursing showing that early workplace participation supports professional socialisation and contextual learning [5, 23]. Overall, these various modalities of delivery demonstrated that, with respect to ECE being flexible and comprehensive, each offered benefits in preparation for students to engage in ECE in the early years of dental education.

Measures of effectiveness across the studies found in this review support the multimodal advantages of ECE. Quantitative measures indicated improvements in knowledge, psychomotor skill, and OSCE/OSPE performance. Chairside demonstration improved understanding [7], while peer-assisted models outperformed faculty led models in terms of experience [8], and guided preclinical exposure facilitated skills as well as empathy [18]. The use of patient-specific 3D-printed teeth was associated with improved crown preparation quality [19], as ECE allowed prosthodontic procedures to be more tangible and understandable [14]. These findings are consistent with evidence from broader health profession such as medical and other allied health sciences education; ECE promotes clinical confidence, psychomotor skill acquisition, the integration of practice and theory, communication and humanistic approaches to patient-centeredness [1, 2, 5, 6]. Comparable trends have also been reported in dental education outside the included studies, where early patient contact supports professional socialisation and clinical preparedness [24, 25]. Together, this empirical evidence supports ECE as a learning platform for students across disciplines that ultimately nurture confidence, competence and transitions to clinical practice.

Experiential outcomes were consistent across the studies included in this review. Students reported high levels of satisfaction, motivation, and confidence following ECE. Bains et al., [26] reported high demand for integration of ECE in the Indian curriculum. More than 90% of students endorsed their academic value to ECE in a report by Maheshwari et al., [7]; and development of psychological safety and reduced anxiety as demonstrated by peer-based strategies [8]. The findings are consistent with medical education and nursing education, where ECE enhances engagement, confidence, professional socialisation emotional readiness, teamwork and learner satisfaction [27, 28]. From all these findings, it can be concluded that ECE is a valid approach to enhance confidence, motivation and preparedness in dental education. However, as these outcomes are largely self-reported, they are to be considered more as an indicator of their educational value than a clear metric of their long-term educational effectiveness in supporting the feasibility of ECE as an acceptable approach to dental educational programs.

The findings of this review are supplemented by qualitative data. Moore et al., [17] illustrated that early mentoring and ECE built professional identity and reduce transition stress, while Ratzmann et al., [16] supplemented the idea of the Greifswald Model as an integrating process between biomedical knowledge and learning with attention to the patient. Studies also highlighted common barriers such as workload, restriction of clinical hours, and organizational challenges [29, 30]. Evidence across medicaland other allied health sciences noticed that, ECE enhance reflective learning, confidence in clinical practice, professional identity, and communication, although these formative experiences may also provoke stress when supervision is delayed, clinical and workload pressures are high, or faculty and time resources are limited [2, 4–6, 31].

The risk of bias to the studies included in this review varied. When using the Risk of Bias 2 tool randomized trials were rated some concern. The risk ratings for randomized controlled trials were limited because of the single centre trial design and short follow-up duration. Half of the non-randomized studies of intervention were rated high risk when considered with the ROBINS-I and other half were rated as some concern. The non-randomized studies of intervention incurred confounding or bias mainly in assessing the outcomes of interest [7, 8]. Qualitative papers were rated as moderate quality using the CASP tool, while the data provided a solid analysis, there was limited applicability [17]. The interrater reliability was good aided by calibration and consensus processes to maintain consistency which were documented (and encouraged transparency). Overall our findings lend to the conservative interpretation of the effect estimates, while taking the quality and factors associated with review, and studies into consideration.

The scarcity of RCT and multicentre studies on ECE in dental education can be attributed to the fact that RCT studies are not scalable and cannot be accommodated within a certain curriculum and the infrastructures of the institution in a multicentre study where every institution can have a separate curriculum. It can be assumed that the above reasons because the dominance of single-centric studies in the dental faculty of the university, even in the context of ECE studies, which require a cautious approach to evaluating their effectiveness. For the designing of the curriculum, it can be assumed that the integration of ECE with a structured approach to the curriculum with defined outcomes and assessments would be necessary, and faculty and accreditation authorities can play a significant role in the improvement of the process with a focus on flexibility and innovation with rigorous evaluation on a universal outcome parameter

### Strengths and limitations

This review is one of the first to fully synthesize evidence on ECE in undergraduate dental education in different contexts, combining quantitative and qualitative results. Its utility is in capturing effectiveness and experience, providing rich cross disciplinary insight. Heterogeneity in study design and outcomes constrained meta-analysis, and conclusions are based on narrative synthesis.

A significant proportion of these studies were entirely dependent on self-assessment outcomes, mainly for experiential components that are prone to social desirability bias and tend to overestimate positive outcomes. While objective measurements (MCQs, OSCE/OSPE scores) were used in some studies [7, 8, 14, 18], there was no uniform usage of objective and long-term measurements of performance. Moreover, a significant proportion of the non-randomized studies were found to have a high risk of bias because of small population samples, lack of control groups, short follow-up durations, and subjective measurement of outcomes. Because of these limitations, it is important to interpret these outcomes with a great degree of caution. Thus, the future research employing robust objective performance measures, multicentre designs, and longitudinal follow-up should be carried out to better establish the educational and clinical impact of ECE in dental education.

Even with these shortcomings, evidence repeatedly favors the inclusion of ECE in dental curricula. Blends of simulation and early patient contact, peer learning, faculty development, and guided support are suggested for effective implementation of ECE on a sustainable basis. Adopting known medical and nursing educational practices can help lead dental education to mainstream ECE as a core part of competency-based curricula.

## Conclusion

This systematic review highlights that, ECE contributes to enhancement ofknowledge and reasoning capabilities, improvement in technical and procedural skills, and instillation of confidence, empathy, inter- and intra-professional communication, and professional identity. Collectively, these outcomes suggest that ECE facilitates a smoother transition for dental students from preclinicalto clinical practice. Future research needs to prioritize well-implemented multicentre and longitudinal studies that utilize standardized and validated measures of outcome to quantify both educational and patient focused impacts. In the meantime, considering curriculum integration of ECE through hybrid models that balance simulation and real patient contact opportunities offer a feasible path forward for preparing competent, confident, and patient-centered dental professionals.

## Data Availability

No datasets were generated or analysed during the current study.
